# Sedentary Behavior and Incident Cancer: A Meta-Analysis of Prospective Studies

**DOI:** 10.1371/journal.pone.0105709

**Published:** 2014-08-25

**Authors:** Dong Shen, Weidong Mao, Tao Liu, Qingfeng Lin, Xiangdong Lu, Qiong Wang, Feng Lin, Ulf Ekelund, Katrien Wijndaele

**Affiliations:** 1 Department of Oncology, The Affiliated Jiangyin Hospital of Southeast University Medical College, Jiangyin, China; 2 Department of Sport Medicine, Norwegian School of Sport Sciences, Oslo, Norway; 3 MRC Epidemiology Unit, Institute of Metabolic Science, University of Cambridge, Cambridge, United Kingdom; West Virginia University, United States of America

## Abstract

**Background:**

Sedentary behavior is ubiquitous in modern adults' daily lives and it has been suggested to be associated with incident cancer. However, the results have been inconsistent. In this study, we performed a systematic review and meta-analysis of prospective cohort studies to clarify the association between sedentary behavior and incident cancer.

**Method:**

PubMed and Embase databases were searched up to March 2014. All prospective cohort studies on the association between sedentary behavior and incident cancer were included. The summary relative risks (RRs) with 95% confidence intervals (CIs) were estimated using random effect model.

**Results:**

A total of 17 prospective studies from 14 articles, including a total of 857,581 participants and 18,553 cases, were included in the analysis for sedentary behavior and risk of incident cancer. The overall meta-analysis suggested that sedentary behavior increased risk of cancer (RR = 1.20, 95%CI = 1.12–1.28), with no evidence of heterogeneity between studies (*I* ^2^ = 7.3%, *P* = 0.368). Subgroup analyses demonstrated that there were statistical associations between sedentary behavior and some cancer types (endometrial cancer: RR = 1.28, 95% CI = 1.08–1.53; colorectal cancer: RR = 1.30, 95%CI = 1.12–1.49; breast cancer: RR = 1.17, 95%CI = 1.03–1.33; lung cancer: RR = 1.27, 95%CI = 1.06–1.52). However, there was no association of sedentary behavior with ovarian cancer (RR = 1.26, 95%CI = 0.87–1.82), renal cell carcinoma (RR = 1.11, 95%CI = 0.87–1.41) or non-Hodgkin lymphoid neoplasms (RR = 1.09, 95%CI = 0.82–1.43).

**Conclusion:**

The present meta-analysis suggested that prolonged sedentary behavior was independently associated with an increased risk of incident endometrial, colorectal, breast, and lung cancers, but not with ovarian cancer, renal cell carcinoma or non-Hodgkin lymphoid neoplasms.

## Introduction

Sedentary behavior is ubiquitous in modern adults' daily lives [1]. It is defined as any waking behavior in a sitting or reclining posture, expending ≤1.5 times the resting energy demand (for example TV viewing, computer use, occupational sitting, reading, and sitting in a car) [2]. Sedentary behavior is distinct from physical inactivity (i.e. not meeting sufficient levels of moderate-to-vigorous physical activity (MVPA)) [2]. The time adults spend sedentary is relatively independent from their time spent in MVPA, for example, individuals may frequently participate in MVPA but still spend substantial amounts of their time sitting [3]. Estimates derived from objective accelerometry suggest that adults spend about 50–60% of their waking day sitting [4]. TV viewing in particular is one of the most prevalent sedentary behaviors, occupying 40% of daily leisure time in some European countries and about 50% in Australia and in the USA [5]. Sedentary behavior may have a detrimental effect on health outcomes, as shown by recent meta-analyses providing evidence that prolonged sedentary behavior increases the risk of the metabolic syndrome [6], type 2 diabetes, cardiovascular disease, and all-cause mortality [7]–[9].

To date, 16 prospective studies have examined the association between sedentary behavior and incident cancer [10]–[23], however, showing inconsistent findings. Although Lynch et al.[24] reviewed the association between sedentary behavior and risk of cancer in 2010, a meta-analysis quantifying the association between this highly prevalent behavior and incident cancer is currently lacking. In addition, 9 new articles related to this topic have been published since 2010 [15]–[23].

In this study, we restricted ourselves to reviewing prospective cohort studies since cross-sectional or case-control studies are subject to recall bias and even reverse causality. Thus, we performed a systematic review and meta-analysis of all published prospective studies to further clarify the association between sedentary behavior and incident cancer.

## Materials and Methods

### Literature and search strategy

The major literature databases including PubMed and Embase were searched. Search terms were (sedentary lifestyle OR sedentary behavior OR sitting time OR watching TV OR TV viewing) and (cancer OR carcinoma OR tumor). The literature search was limited to English language. The literature search was updated on March 4, 2014.

### Inclusion criteria and data extraction

Studies included in the meta-analysis met all the following inclusion criteria: (1) evaluated the association between sedentary behavior (total sitting time, occupational sitting time, leisure sitting time or TV viewing) and incident cancer; (2) used a prospective cohort design; (3) provided relative risks (RRs) or hazard ratios (HRs) with 95%CIs for highest versus lowest level of sedentary behavior. The following information was extracted from each study: (1) the first author; (2) publication year; (3) country name; (4) sex distribution; (5) age distribution of study population at baseline; (6) average duration of follow-up; (7) number of cases and study population; (8) types of cancer; (9) RR or HR with 95%CI for highest versus lowest level of sedentary behavior; (10) covariates used in adjustment. Two authors independently assessed the articles for compliance with the inclusion/exclusion criteria and resolved disagreements through discussion.

### Statistical analysis

Random [25] effects model was used to calculate pooled RRs with 95%CIs for the highest versus the lowest level of sedentary behavior. Sensitivity analysis, after exclusion one study at each time, was applied to test the stability of the results. Subgroup analyses were performed to investigate the association between sedentary behavior and risk of types of cancer. In addition, we also tested the association between TV viewing and risk of cancer. Publication bias was assessed by Begg's test [26] and Egger's test [27] (*P*<0.05 was considered statistically significant). Statistical analysis was conducted using STATA version 11 (StataCorp LP, College Station, Texas, USA).

## Results

### Characteristics of included prospective studies

**Figure 1:**
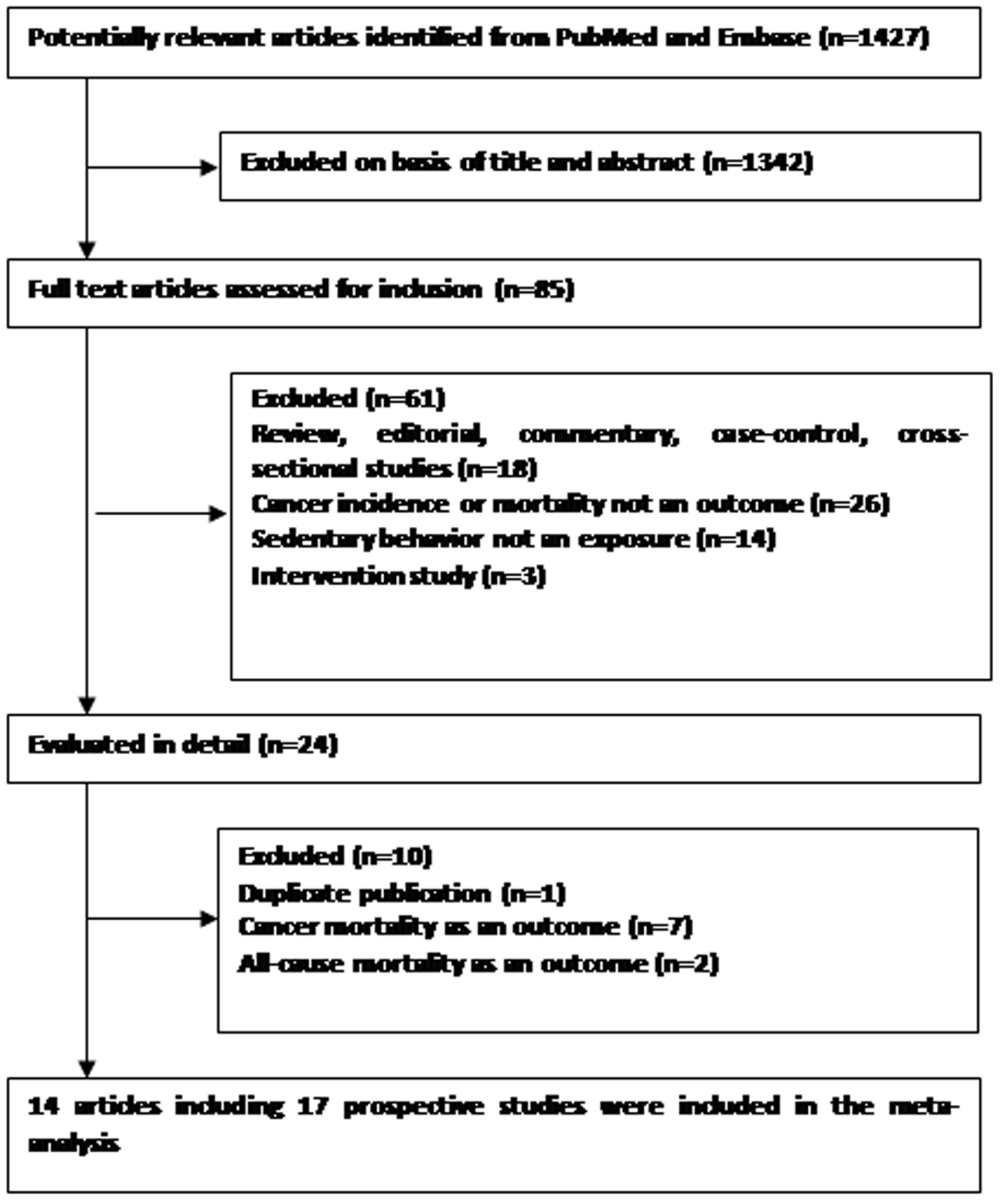
Flow diagram of literature search and study selection.

Following the literature search and selection, a total of 17 prospective studies from 14 publications were included in the meta-analysis examining the association between sedentary behavior and risk of incident cancer (Figure 1). The duration of follow-up ranged from 3.8 to 16 years. 13 studies originated from America, 2 study from Europe, and 2 studies from East Asia. The characteristics of included prospective studies are listed in Tables 1 and 2.

**Table 1:** Characteristics of included prospective studies examining the association between sedentary behavior and incident cancer.

Study	Country	Sex	Age at baseline (years)	No. of participants	No. of cases	Duration of follow-up (years)	Cancer site	Sedentary measure
Friberg et al, 2006 [10]	Sweden	Women	50–83	33723	199	7.2	Endometrial cancer	Watching TV/sitting
Patel et al, 2006 [11]	US	Women	50–74	59695	314	9	Ovarian cancer	Total sitting time (watching TV, reading, etc)
Patel et al, 2008 [12]	US	Women	50–74	42672	466	11	Endometrial cancer	Total sitting time (watching TV, reading, etc)
Howard et al, 2008 [13]	US	Men	50–71	292069	3240	6.9	Colorectal cancer	Watching TV/videos; Total sitting time
		Women	50–71	196651	1482	6.9	Colorectal cancer	Watching TV/videos; Total sitting time
Gierach et al, 2009 [14]	US	Women	50–71	109621	1052	3.8	Endometrial cancer	Watching TV/videos; Total sitting time
George et al, 2010 [15]	US	Women	50–71	97039	3436	7	Breast cancer	Watching TV/videos; Total sitting time
George et al, 2011 [16]	US	Men and women	50–71	289512	1206	10	Renal cell carcinoma	Watching TV/videos; Total sitting time
Pronk et al, 2011 [17]	China	Women	40–70	73049	717	9	Breast cancer	Total sitting time
Teras et al, 2012 [18]	US	Women	50–74	77001	863	15	Non-Hodgkin lymphoid neoplasm	Leisure sitting time
		Men	50–74	69849	1139	15	Non-Hodgkin lymphoid neoplasm	Leisure sitting time
Cohen et al, 2013 [19]	US	Women	40–79	2730	546	9	Breast cancer	Total sitting time (watching TV/movies, using a computer at home, reading, sitting at work)
Lam et al, 2013 [20]	US	Men and women	50–71	158415	532	11	Lung cancer	Watching TV/videos; Total sitting time
Simons et al, 2013 [21]	Netherlands	Men	55–69	58251	1819	16	Colorectal cancer	Occupational sitting time
Ukawa et al, 2013 [22]	Japan	Men	40–79	23090	589	15.6	Lung cancer	Watching TV
		Women	40–79	31168	200	15.6	Lung cancer	Watching TV
Xiao et al, 2013 [23]	US	Women	50–71	148892	753	11	Ovarian cancer	Watching TV/videos; Total sitting time

**Table 2:** RRs and 95%CIs reported by included prospective studies examining the association between sedentary behavior and incident cancer.

Study	Outcome	Sedentary measure	Sedentary category and RR (95%CI)	Adjusted confounders
Friberg et al, 2006 [10]	Endometrial cancer	Watching TV/sitting	<5 h/d: 1 (Referent); ≥5 h/d: 1.66 (1.05–2.61)	Age, parity, history of diabetes, total fruit and vegetable, education, and work/occupation, walking/bicycling, household work, leisure time activity, and body mass index
Patel et al, 2006 [11]	Ovarian cancer	Total sitting time (watching TV, reading, etc)	<3 h/d: 1 (Referent); 3–5 h/d: 1.21 (0.95–1.54); ≥6 h/d: 1.55 (1.08–2.22)	Age, race, body mass index, family history of breast and/or ovarian cancer, age at menopause, age at menarche, oral contraceptive use, parity, hysterectomy, and postmenopausal hormone replacement therapy use
Patel et al, 2008 [12]	Endometrial cancer	Total sitting time (watching TV, reading, etc)	<3 h/d: 1 (Referent); 3–5 h/d: 1.02 (0.83–1.25); ≥6 h/d: 1.18 (0.87–1.59)	Age, age at menarche, age at menopause, duration of OC use, parity, smoking, total caloric intake, personal history of diabetes, postmenopausal HT use, and body mass index.
Howard et al, 2008 [13]	Colorectal cancer (men)	Watching TV/videos	<3 h/d: 1 (Referent); 3–4 h/d: 1.14(1.00–1.30); 5–6 h/d: 1.22(1.03–1.45); 7–8 h/d: 1.15(0.81–1.63); ≥9 h/d:1.56 (1.11–2.20)	Age, smoking, alcohol consumption, education, race, family history of colon cancer, total energy and energy-adjusted intake of red meat, calcium, whole grains, fruit and vegetables, total physical activity and body mass index.
		Total sitting time	<3 h/d: 1 (Referent); 3–4 h/d: 1.20(1.01–1.43); 5–6 h/d: 1.21(1.02–1.44); 7–8 h/d:1.23(1.01–1.50); ≥9 h/d:1.22 (0.96–1.55)	
	Colorectal cancer (women)	Watching TV/videos	<3 h/d: 1 (Referent); 3–4 h/d: 0.94(0.78–1.13); 5–6 h/d:1.03 (0.82–1.30); 7–8 h/d:1.04(0.68–1.58); ≥9 h/d:1.45 (0.99–2.13)	Age, smoking, alcohol consumption, education, race, family history of colon cancer, total energy and energy-adjusted intake of red meat, calcium, whole grains, fruit and vegetables, total physical activity, menopausal hormone therapy, and body mass index.
		Total sitting time	<3 h/d: 1 (Referent); 3–4 h/d: 0.96 (0.77–1.19); 5–6 h/d:1.04 (0.84–1.30); 7–8 h/d:0.96(0.73–1.26); ≥9 h/d:1.23 (0.89–1.70)	
Gierach et al, 2009 [14]	Endometrial cancer	Watching TV/videos	<3 h/d: 1 (Referent); 3–4 h/d: 1.11 (0.92–1.33); 5–6 h/d:1.08 (0.86–1.37); ≥7 h/d:1.21 (0.87–1.67)	Age, race, smoking status, parity, ever use of oral contraceptives, age at menopause, and hormone therapy formulation and body mass index
		Total sitting time	<3 h/d: 1 (Referent); 3–4 h/d: 1.07 (0.85–1.37); 5–6 h/d:1.31 (1.04–1.65); ≥7 h/d:1.26 (0.99–1.62)	
George et al, 2010 [15]	Breast cancer	Watching TV/videos	For invasive breast cancer: <3 h/d: 1 (Referent); 3–4 h/d: 1.00 (0.92–1.09); 5–6 h/d: 0.93 (0.83–1.05); 7–8 h/d: 1.04 (0.84–1.30); ≥9 h/d: 1.12 (0.89–1.41)	Age, energy intake, recreational moderate–vigorous physical activity, parity or age at first live birth, menopausal hormone therapy use, number of breast biopsies, smoking, alcohol intake in grams per day, race, education and body mass index
			For situ breast cancer: <3 h/d: 1 (Referent); 3–4 h/d: 1.16 (0.95–1.41); 5–6 h/d: 1.32 (1.03–1.71); 7–8 h/d: 1.50 (0.95–2.38); ≥9 h/d: 1.01 (0.56–1.83)	
		Total sitting time	For invasive breast cancer: <3 h/d: 1 (Referent); 3–4 h/d: 1.07 (0.96–1.19); 5–6 h/d: 1.08 (0.97– 1.20); 7–8 h/d: 1.08 (0.95–1.23); ≥9 h/d: 1.08 (0.92–1.27)	
			For situ breast cancer: <3 h/d: 1 (Referent); 3–4 h/d: 1.14 (0.89–1.46);5–6 h/d: 1.24 (0.97– 1.59); 7–8 h/d: 1.17 (0.88–1.57); ≥9 h/d: 1.12 (0.78–1.61)	
George et al, 2011 [16]	Renal cell carcinoma	Watching TV/videos	<1 h/d: 1 (Referent); 1–2 h/d:1.06 (0.81–1.39); 3–4 h/d:1.15 (0.88–1.49); 5–6 h/d:1.15 (0.86–1.53); ≥7 h/d:0.96 (0.66–1.38)	Age, sex, race, history of diabetes, smoking, alcohol intake, diet quality, energy intake, and recreational moderate-vigorous physical activity
		Total sitting time	<3 h/d: 1 (Referent); 3–4 h/d: 1.20 (1.02–1.42); 5–6 h/d: 1.02 (0.86–1.21); 7–8 h/d:1.04 (0.85–1.27); ≥9 h/d:1.11 (0.87–1.41)	
Pronk et al, 2011 [17]	Breast cancer	Total sitting time	≥4 h/d: 1 (Referent); 3.69–4 h/d: 0.92 (0.57–1.50); 1.2–3.69 h/d: 1.20–3.69 h/d: 0.82 (0.67–1.00); <1.2 h/d: 0.81(0.65– 1.01)	Age, education, family history of breast cancer, age at first birth, and number of pregnancies
Teras et al, 2012 [18]	Non-Hodgkin lymphoid neoplasm (women)	Leisure sitting time	<3 h/d: 1 (Referent); 3–5 h/d: 1.19 (1.03–1.37); ≥6 h/d: 1.26 (1.01–1.59)	Age at baseline, family history of hematopoietic cancer, education, smoking status, alcohol intake, body mass index, height and physical activity
	Non-Hodgkin lymphoid neoplasm (men)	Leisure sitting time	<3 h/d: 1 (Referent); 3–5 h/d: 1.00 (0.88–1.13); ≥6 h/d: 0.95 (0.79–1.15)	
Cohen et al, 2013 [19]	Breast cancer	Total sitting time (watching TV/movies, using a computer at home, reading, sitting at work)	<5.5 h/d: 1 (Referent); 5.5–8.1 h/d: 1.29 (0.94–1.77); 8.2–11.9 h/d1.25(0.90–1.73); ≥12 h/d:1.41 (1.01–1.95)	Matching factors (age, race, menopausal status, and enrollment source) were accounted for in the conditional analysis. Additional covariates included in the models were education, household income, body mass index at age 21 years, cigarette smoking, ever use of hormone replacement therapy, parity, age at menarche, first-degree family history of breast cancer, having health insurance and total activity
Lam et al, 2013 [20]	Lung cancer	Watching TV/videos	<3 h/d: 1 (Referent); 3–4 h/d: 1.16 (0.91–1.48); ≥5 h/d:1.06 (0.77–1.46)	Age, current body mass index, education, ethnicity, vigorous activity, alcohol consumption, total caloric intake.
		Total sitting time	<3 h/d: 1 (Referent); 3–4 h/d: 1.36 (1.00–1.85); ≥5 h/d:1.28 (0.96–1.72)	
Simons et al, 2013 [21]	Colorectal cancer	Occupational sitting time	6–8 h/d: 1 (Referent); 2–6 h/d: 0.74 (0.61– 0.89); <2 h/d: 0.72 (0.58–0.89)	Age, family history of colorectal cancer, smoking status, alcohol intake, body mass index, meat intake, processed meat intake, and total energy intake
Ukawa et al, 2013 [22]	Lung cancer (men)	Watching TV	<2 h/d: 1 (Referent); 2–4 h/d: 1.24 (0.98–1.60); ≥4 h/d: 1.36 (1.04–1.80)	Age, body mass index, education, marital status, alcohol drinking, smoking status, intake of green leafy vegetables, oranges, and fruits other than oranges
	Lung cancer (women)	Watching TV	<2 h/d: 1 (Referent); 2–4 h/d: 1.11 (0.76–1.67); ≥4 h/d: 1.03 (0.67–1.62)	Age, body mass index, education, marital status, alcohol drinking, smoking status, intake of green leafy vegetables, oranges, and fruits other than oranges
Xiao et al, 2013 [23]	Ovarian cancer	Watching TV/videos	<3 h/d: 1 (Referent); 3–4 h/d: 0.96 (0.78–1.18); 5–6 h/d: 0.80 (0.59–1.07); ≥7 h/d: 1.02 (0.67–1.55)	Age, no. of live birth, age at menarche, age at menopause, race, education, marital status, oral contraceptive use, MHT use, and smoking
		Total sitting time	<3 h/d: 1 (Referent); 3–4 h/d: 0.90 (0.69–1.16); 5–6 h/d:0.85 (0.65–1.10); ≥7 h/d:1.06 (0.81–1.39)	

### Sedentary behavior and incident cancer

**Figure 2:**
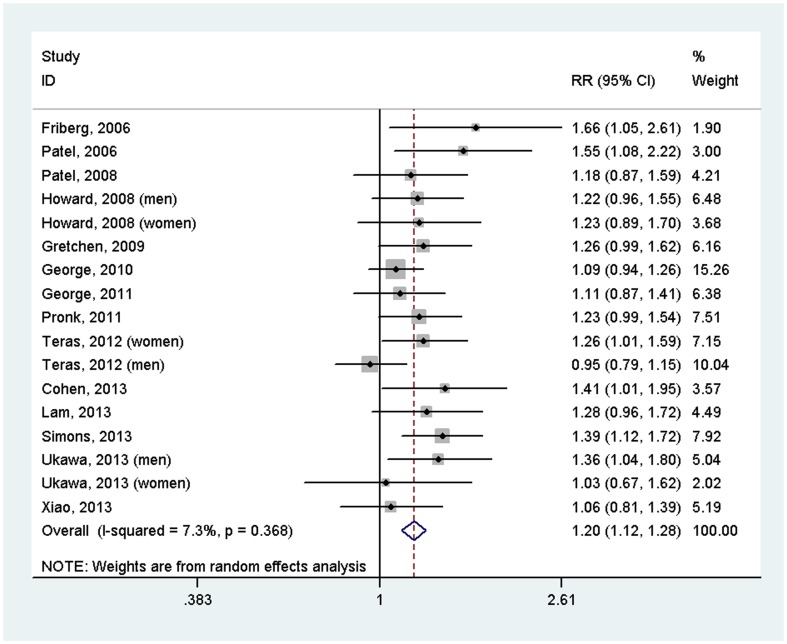
Forest plot of the association between sedentary behavior and risk of incident cancer (highest versus lowest level).

17 prospective studies from 14 articles were included in the meta-analysis, including a total of 857,581 participants and 18,553 cases. The overall meta-analysis suggested that sedentary behavior increased risk of cancer (RR = 1.20, 95%CI = 1.12–1.28), with no evidence of heterogeneity between studies (*I* ^2^ = 7.3%, *P* = 0.368) (Figure 2). Sensitivity analysis suggested that the result was stable, with ORs and 95%CIs ranging from 1.18 (1.11–1.26) to 1.22 (1.15–1.31). There was no publication bias (*P* = 0.202). It should be noted that RRs and 95% CIs with adjustment for potential confounding factors, such as BMI, physical activity and energy intake, from all included studies were pooled together using meta-analysis.

**Figure 3:**
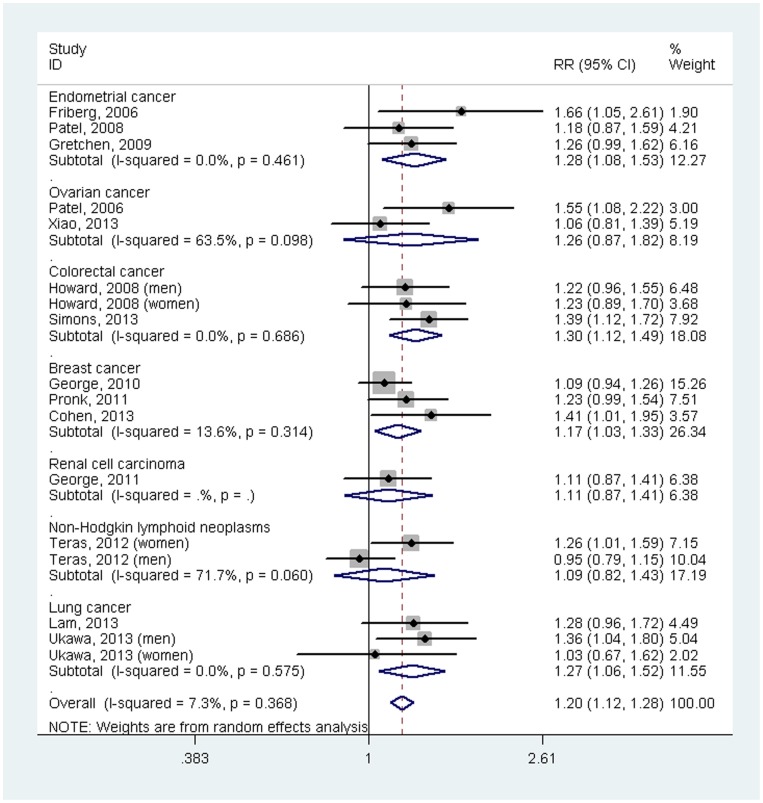
Forest plot of the association between sedentary behavior and risk of incident cancer by cancer site (highest versus lowest level).

In the subgroup analyses (Figure 3), there were statistical associations between sedentary behavior and some cancer types (endometrial cancer: RR = 1.28, 95% CI = 1.08–1.53; colorectal cancer: RR = 1.30, 95%CI = 1.12–1.49; breast cancer: RR = 1.17, 95%CI = 1.03–1.33; lung cancer: RR = 1.27, 95%CI = 1.06–1.52). However, there was no association of sedentary behavior with ovarian cancer (RR = 1.26, 95%CI = 0.87–1.82), renal cell carcinoma (RR = 1.11, 95%CI = 0.87–1.41) or non-Hodgkin lymphoid neoplasms (RR = 1.09, 95%CI = 0.82–1.43).

**Figure 4:**
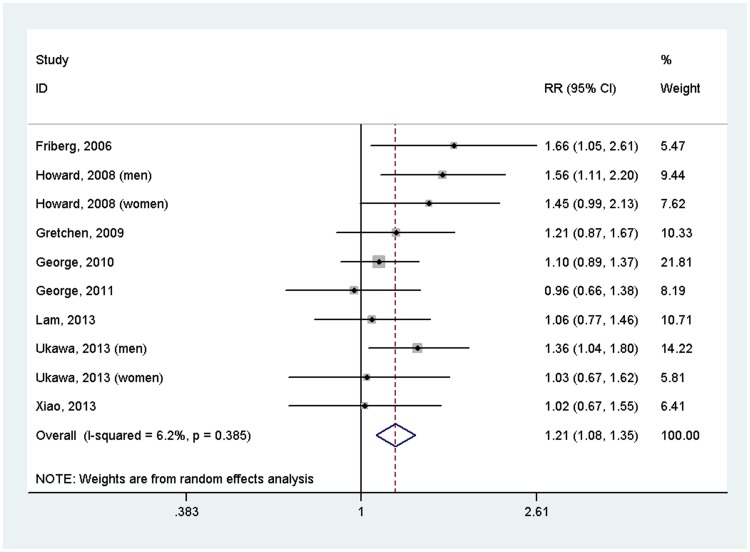
Forest plot of the association between TV viewing and risk of incident cancer (highest versus lowest level).

Since TV viewing is the main type of sedentary behavior, we also investigated the association between TV viewing and risk of cancer. The results suggested that sedentary behavior increased the risk of cancer (RR = 1.21, 95%CI = 1.08–1.35) (Figure 4). We did not performed subgroup analyses based on type of cancer again because of limited studies for each cancer type.

## Discussion

Our meta-analyses suggest that prolonged sedentary behavior is associated with an increased risk of some cancer types including colorectal, lung, breast, and endometrial cancers, but not with ovarian cancer, renal cell carcinoma or non-Hodgkin lymphoid neoplasms. The positive association was independent of traditional risk factors including BMI, physical activity and energy intake.

In many Western countries, adults spend large proportions of their awake time sedentary. It is estimated that the average US population spends about 35 h/week watching TV, 2 h/week watching time-shifted TV, and 4 h/week on the internet [28]. Although insufficient physical activity has long been considered as a risk factor of many chronic diseases (e.g., type 2 diabetes [29], coronary heart disease [30], cancer [31]) and all-cause mortality [32], examining the independent relationships between sedentary time and health outcomes is fairly recent. In 2011, a meta-analysis performed by Grøntved and Hu [7] reported that each 2-hour increment of TV viewing daily was associated with increased risk of type 2 diabetes (RR 1.20, 95%CI = 1.14–1.27), cardiovascular disease (RR 1.15, 95%CI = 1.06–1.23), and all-cause mortality (RR = 1.13, 95%CI 1.07–1.18). Another meta-analysis by Ford and Caspersen [9] demonstrated a RR of 1.05 (95%CI = 1.01–1.09) for every 2-hour/day increase for the association between sedentary behavior and cardiovascular events. Most recently, Cong et al. [33] reported that sedentary behavior was associated with an increased risk of colon cancer. However, their conclusion is mainly based on case-control studies and also they did not examine the association between sedentary behavior and risk of other types of cancer.

Several plausible mechanisms may explain the observed association between sedentary behavior and risk of some cancers. First, when a substantial amount of time is spent sitting, especially in front of the TV, this automatically means less physical activity, and it may lead to a higher energy intake [34], both which affect energy balance in such a way that possibly overweight/obesity results. However, the observed associations remained when adjusting for BMI or WC, energy intake, and physical activity. In addition, BMI or WC may then act more like a confounder. As is known, obese individuals are more prone to stay sedentary than non-obese ones. Second, excessive sitting time could increase levels of inflammatory factors such as tumor necrosis factor-α, interleukin-6, and leptin which are known risk predictors for cancer [35]. Third, sitting time has been hypothesized to influence sex hormones which could affect immune function [24].

Strengths of our meta-analysis include the prospective design, large sample size, long duration of follow-up, and ability to control for many potential confounding factors for the included studies. However, several limitations should be considered. First, sedentary behavior time in all included studies was self-reported, which could have resulted in the possibility of exposure misclassification and thereby underestimation of the true association between sedentary behavior and risk of cancer. Second, sedentary behavior was only measured at baseline for most included studies, and individuals may have changed their sedentary lifestyle during follow-up. This misclassification may also attenuate the true association. Third, although many potential confounding factors have been adjusted for, residual confounding because of poorly measured or unmeasured confounding factors may influence our results, particularly also for MVPA, a key confounding variable [36]. In addition, we can not rule out residual confounding of smoking on lung cancer. Fourth, we were unable to distinguish between most types of sitting (i.e., TV viewing, reading, using computer, sitting at work) which may have different levels of energy expenditure or be associated with different underlying confounding structures. Fifth, there was limited number of studies for each cancer site. However, the total number of cancer cases for each cancer site was relatively large (all *n*>1000 for each cancer site). Sixth, the included 17 studies adjusted for different confounding factors, which might have influenced the results.

In this study, we found significant associations between sedentary behavior and risk of some types of cancer including colorectal, lung, breast, and endometrial cancers. Public health guidelines for prevention and control of incident cancer may need to consider recommendations about reducing time spent sitting in addition to increasing MVPA. Actually, World Cancer Research Fund report has already made a statement on the importance of limiting sedentary behavior. Our current findings based on prospective studies further supported this statement.

## Supporting Information

Checklist S1 PRISMA checklist.
(DOC)
